# Improving Risk Prediction of Methicillin-Resistant Staphylococcus aureus Using Machine Learning Methods With Network Features: Retrospective Development Study

**DOI:** 10.2196/48067

**Published:** 2024-05-16

**Authors:** Methun Kamruzzaman, Jack Heavey, Alexander Song, Matthew Bielskas, Parantapa Bhattacharya, Gregory Madden, Eili Klein, Xinwei Deng, Anil Vullikanti

**Affiliations:** 1 University of Virginia Charlottesville, VA United States; 2 Division of Infectious Diseases & International Health Department of Medicine University of Virginia School of Medicine Charlottesville, VA United States; 3 Department of Emergency Medicine Johns Hopkins School of Medicine Baltimore, MD United States; 4 Center for Disease Dynamics, Economics and Policy Washington, DC, DC United States; 5 Department of Statistics Virginia Tech Blacksburg, VA United States; 6 Department of Computer Science University of Virginia Charlottesville, VA United States

**Keywords:** methicillin-resistant Staphylococcus aureus, network, machine learning, penalized logistic regression, ensemble learning, gradient-boosted classifier, random forest classifier, extreme boosted gradient boosted classifier, Shapley Additive Explanations, SHAP, health care–associated infection, HAI

## Abstract

**Background:**

Health care–associated infections due to multidrug-resistant organisms (MDROs), such as methicillin-resistant *Staphylococcus aureus* (MRSA) and *Clostridioides difficile* (CDI), place a significant burden on our health care infrastructure.

**Objective:**

Screening for MDROs is an important mechanism for preventing spread but is resource intensive. The objective of this study was to develop automated tools that can predict colonization or infection risk using electronic health record (EHR) data, provide useful information to aid infection control, and guide empiric antibiotic coverage.

**Methods:**

We retrospectively developed a machine learning model to detect MRSA colonization and infection in undifferentiated patients at the time of sample collection from hospitalized patients at the University of Virginia Hospital. We used clinical and nonclinical features derived from on-admission and throughout-stay information from the patient’s EHR data to build the model. In addition, we used a class of features derived from contact networks in EHR data; these network features can capture patients’ contacts with providers and other patients, improving model interpretability and accuracy for predicting the outcome of surveillance tests for MRSA. Finally, we explored heterogeneous models for different patient subpopulations, for example, those admitted to an intensive care unit or emergency department or those with specific testing histories, which perform better.

**Results:**

We found that the penalized logistic regression performs better than other methods, and this model’s performance measured in terms of its receiver operating characteristics-area under the curve score improves by nearly 11% when we use polynomial (second-degree) transformation of the features. Some significant features in predicting MDRO risk include antibiotic use, surgery, use of devices, dialysis, patient’s comorbidity conditions, and network features. Among these, network features add the most value and improve the model’s performance by at least 15%. The penalized logistic regression model with the same transformation of features also performs better than other models for specific patient subpopulations.

**Conclusions:**

Our study shows that MRSA risk prediction can be conducted quite effectively by machine learning methods using clinical and nonclinical features derived from EHR data. Network features are the most predictive and provide significant improvement over prior methods. Furthermore, heterogeneous prediction models for different patient subpopulations enhance the model’s performance.

## Introduction

Multidrug-resistant organisms (MDROs), such as *Clostridioides difficile* (CDI), multidrug-resistant gram-negative bacteria (carbapenem-resistant *Acinetobacter baumannii* and carbapenem-resistant Enterobacterales), methicillin-resistant *Staphylococcus aureus* (MRSA), and vancomycin-resistant enterococci, are among the top 10 threats to global health [1]. Health care–associated infections (HAIs) due to MDROs are associated with increased complications, longer hospital stays, and increased mortality. For example, Weiner-Lastinger et al [2] report that HAIs have resulted in billions of dollars in increased healthcare costs [3]. MRSA is one of the most common causes of HAIs and a serious antimicrobial resistance threat, responsible for >10,000 deaths a year in the United States alone [4]. Similar to many other MDROs, MRSA can be easily spread in a hospital from hospitalized patients via contact with the health care environment (ie, shared patient rooms) and health care workers.

Antimicrobial stewardship, which seeks to optimize antibiotic treatment regimens, and infection prevention and control, which involves monitoring, investigating, and managing factors related to MDRO transmission, are the main tools for mitigating the risks of acquisition and severe outcomes of MDROs [5]. Surveillance testing is a critical component of both antimicrobial stewardship and infection prevention control. However, testing is expensive and slow; current laboratory procedures typically require at least 72 hours to report MRSA found in a patient’s culture [6]. The delay in testing results in three problems in the hospital: (1) colonized patients remain undetected, leading to potential spread; (2) clinicians treat infections empirically; and (3) increased resource use for contact precautions, leading to both over- and undertreatment.

While several different studies have examined MRSA risk prediction (eg, [6-13]), none to date have progressed to clinical practice due to limitations in generalizability, sample size, and imbalanced data (these are discussed further in the Discussion section). In this study, we demonstrate how improving the hospital context, particularly how patients are connected, can improve the performance of machine learning methods for predicting the outcomes of MRSA surveillance tests, using a rich set of clinical and nonclinical features derived from on-admission and throughout-stay information from a large electronic health record (EHR) data set for patients admitted to the University of Virginia (UVA) Hospital.

## Methods

### Data Set

We used patient data from the UVA Hospital during 2010-2022. Overall, 27,612 patients in the dataset were tested for MRSA, and 4171 (15.11%) of them were positive; these patients had 37,237 hospital encounters. The data of each patient’s visit can be separated into two parts: (1) on-admission data and (2) clinical event or throughout-stay data, which we have described here:

*On-admission* data consist of patient demographics and visit information. Patient demographics include information about age, gender, race, ethnicity, country, and state. Visit information includes admission and discharge dates, admission source, admission type, and discharge destination.

Clinical event data represent information collected during the visit. We considered the following event data:

- Procedure: it includes the following kinds of events during this visit or at any time 90 days before this visit: (1) surgeries, (2) device implant or replacement, and (3) dialysis. For a visit, no data after the test collection are used.
- Medication: as MRSA is resistant to specific antibiotics, we also examined prior antibiotic use. We computed the *Days on Therapy*, which indicates whether a patient takes any antibiotic on any specific day. This feature also calculates whether a patient took any antibiotic in the last 90 days of this hospital visit.
- Comorbidity: the International Classification of Diseases, Tenth Revision, code of a patient, which is collected from that patient’s medical history, is used to pull comorbidity information using the comorbidity package in R programming language (R Foundation for Statistical Computing). Both Charlson and Elixhauser scores are pulled. It involves other physical conditions such as diabetes, a history of stroke, and a history of dementia.
- MRSA laboratory test: we included both (1) clinical cultures and blood, respiratory, and urine samples collected as part of routine care, which typically requires 48 to 72 hours to return results, and (2) polymerase chain reaction (PCR) surveillance tests, which are administered to MRSA-negative patients admitted to an intensive care unit (ICU; per current hospital policy) or per physician request and typically return results in <72 hours. While surveillance tests provide positive and negative results, clinical cultures may be sent from specimens that are not expected to yield MRSA, even in the presence of an active MRSA infection; therefore, a negative clinical culture result is not considered a definite indicator of noninfection. The nares MRSA PCR likely has equal or higher sensitivity than the nares culture for MRSA [14]. We noted that, in general, testing is not completely unbiased (a patient with an MRSA-positive result admitted to an ICU would not technically need to be screened if they are already on precautions), which might impact the quality of the data set and the results, as we discuss later in the Discussion section.

We applied state-of-the-art machine learning methods to predict the risk of MRSA infection at a given time for a patient, modeled by the outcome of a surveillance test. The data set is split into training (80%) and testing (20%) portions. The model is estimated using the training data, and the hyperparameters are chosen by cross-validation. There are many metrics to evaluate model performance. We used receiver operating characteristics-area under the curve (ROC-AUC) as the overall performance metric of the model (the model evaluation metrics are described in Multimedia Appendix 1), and a higher value is better. For clinicians, an important objective is to reduce the number of false-negative cases. Therefore, we also used the *false negative rate* 
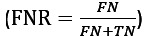
to evaluate the model performance, with a lower value indicating a lower false-negative prediction. The overall model performance is proportional to the ROC-AUC score and inversely proportional to the FNR score.

### Problem Statement

The d-days ahead model’s MRSA test prediction problem: using features defined from the patient EHR data till some time (*t’ = t – d*) predict the outcome of an MRSA surveillance test performed at time *t*. Formally, let *x(t’)* denote a feature vector for a patient defined till time *t* and let *y(t)* denote the result of an MRSA surveillance test performed at time *t*. The objective is to predict if y(t) = 1 using *x(t’)*.

The specific questions we study are as follows:

1. How well can MRSA surveillance test results be predicted? What machine learning methods perform well, and what features are the most predictive?
2. Are better predictions possible for specific, meaningful subpopulations?
3. How does the performance vary with *d*?
4. Does training with a biased data set (as performed in previous work) impact the true performance?

### Interesting Features

Several risk factors for MRSA have been identified in previous studies [15,16]: (1) hospitalization within the past 6 to 12 months, (2) residing in a chronic care facility, (3) being a health care worker, (5) being an intravenous drug user, (5) frequent antibiotic use, (6) antimicrobial therapy within 1 year, (7) history of endotracheal intubation, (8) underlying chronic disorder, (9) presence of an indwelling venous or urinary catheter, (10) history of any surgical procedure, (11) household contact with an identified risk factor, and (12) hypoalbuminemia. We extracted all the aforementioned features from the UVA data set. We created patient-patient and patient-provider interaction networks and extracted the following features from those networks. In addition, we derived many features based on the existing features described in the subsequent section. The total number of features is 108, and the MRSA test outcome is the target feature.

**Figure 1:**
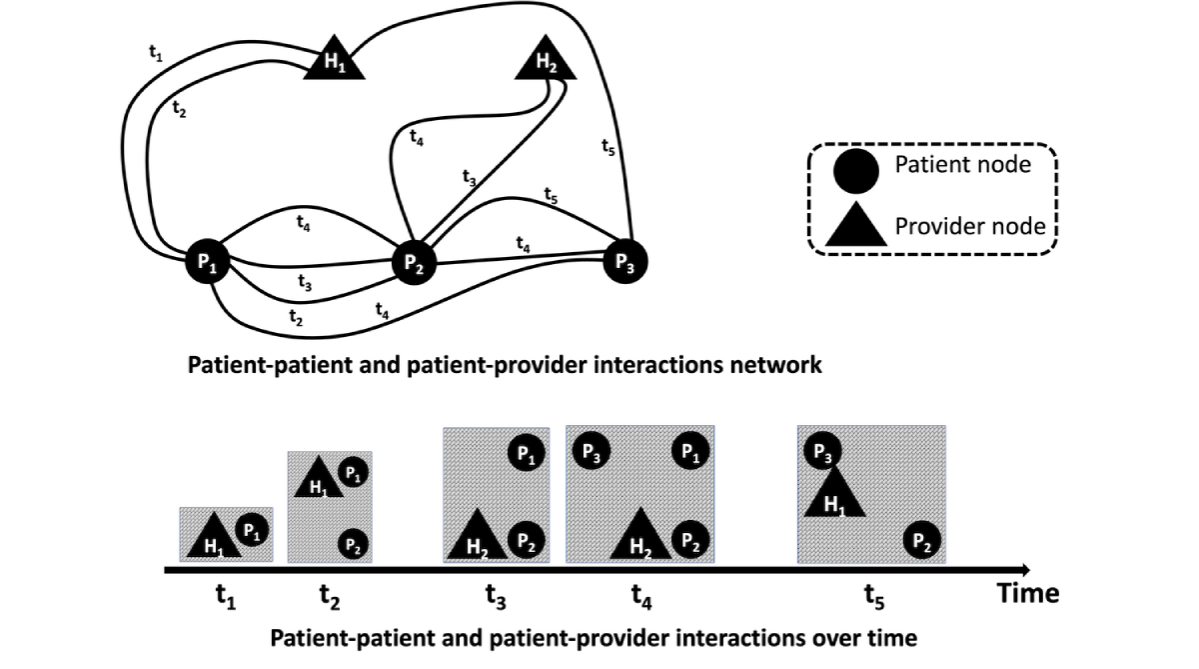
Patient-patient and patient-provider interactions are shown on the timeline, where each box represents a room in the hospital, patients are indicated by circles (marked with P) and health care providers are indicated by triangles (marked with H). Multiple patients could share a room, and a provider might visit multiple patients over time. A network is constructed from these interaction events over time. If 2 patients share a room for a certain period (at least for 15 min), we construct an edge between the corresponding patient nodes; similarly, if a provider visits a patient for a certain period (at least for 15 min), we construct an edge between the corresponding patient and provider nodes.

1. Network features: we constructed a contact network *G = (V, E)* (as shown in Figure 1), in which we have patient nodes *u_p_ ∈ V* for each patient *p* and a provider node *u_h_ ∈ V* for each provider *h*. An edge or contact *(u_p1_, u_p2_)* ∈ E between 2 patient nodes u_p1_ and u_p2_ indicates that both patients p_1_ and p_2_, respectively, were colocated (share a common space, a hospital unit in our case) for at least a certain period, in this case at least 900 seconds. Similarly, we defined patient-provider contacts. For instance, in Figure 1, patient P_1_ and provider H_1_ are colocated at time t_1_, which is represented as edge (u*_p1_*, u*_h1_*). The #provider incidents on patient P_1_ in the time interval [*t_1_, t_2_*] is 2, whereas in the time interval [*t_1_, t_3_*], it is 3. We did not use the number of patients and providers that a patient comes into direct contact with as a feature. Instead, we defined slightly different features based on contacts during a time interval, which we found to be more predictive. We take time to be in days. On the basis of the number of contacts for a patient *p* or a provider *h* over a period, we constructed the following features:

- *MRSA α*: for a patient p, S_p,t_(α) = {p’: (u_p_, u_p’_) *∈ E, p’ is labeled positive at time t’ ∈ {t – a, t]}*, denotes the set of patients who came in contact with *p* and tested positive in the last α days. We refer to |S_p,t_(α)| as MRSA α.
- Provider β: for a patient p, §_p,t_ (β) = {h: (u_p_, u_h_) *∈ E, h visited p at time t’ ∈ (t – β, t]}*. We refer to |§_p,t_ (β)| as Provider β.
- MRSA positive patients collocated with the patient *l*: at the UVA Hospital, patients with an MRSA-positive result might be “cohorted,” that is, they might share a room because they have similar precautions to improve occupancy. For a patient *p*, let ƒ_p,t_(u, γ) = {p’:(u_p_, u_p’_) ∈ E, p’ is labeled positive at t’ *∈ (t’ – γ,t]* and is in the hospital unit u with p}. We referred to |ƒ_p,t_(u,γ)| as the number of patients with colocated MRSA.
- *Bed reuse* Π: let Π_p,t_(x) = {p’: (u_p_, u_p’_) ∉ E, p’ is labeled positive at time t’<t and stayed in the same bed *x*}. We refer to | Π_p,t_(x)| as the number of times Bed *x* reuse.

Note that all of the aforementioned features are defined for a particular time, t. Therefore, MRSA *α* and other features should be indexed by the patient and time. To avoid notational clutter, we omit them here when they are clear from the context. For example, suppose t_1_=1, t_2_=2, t_3_=3, t_4_=4, and t_5_=5, as shown in Figure 1. Suppose patient P_2_ is tested positive at time 4. Then, for patient P_1_, we would have “MRSA 2” at time t=5 equal to 1, but “MRSA 2” at time t=3 equals 0. For patient P_2_, Provider 2 at time t=2 is 0, but Provider 2 at time t=3 is 1.

2. Length of stay: for patients *p* in a hospital encounter, let *t_1_* denote the admission time and *t* denote the MRSA test time. The corresponding length of hospital stay (before the MRSA test) was computed as t–t1. For the d-days (d ≥ 0) ahead model, we computed the corresponding length of stay (before the MRSA test) as max{t-d-t_1_, 0}. Note that t-d-t_1_ could be negative if the patient has not been in the hospital long enough—in this case, we took the length of stay to be 0.

3. From the health care facility is a Boolean feature that indicates whether the patient is admitted to the hospital from either “skilled nursing, intermediate care, or assisted living facility” or “long term acute care hospital.” For the d-days ahead model, the feature is defined to be 0 if *t_1_*-d<0, where *t_1_* is the admission date, and 1, otherwise.

4. δ days observation: we construct several Boolean features based on events in the last δ days before an MRSA test time. For a patient p in a hospital encounter, let T(e) denote the set of times for a specific event e. We defined Boolean variable e_δ_(t)={∃_t1_, t1∈T(e), t_1_<t, 0≤(t-t_1_)≤δ}. We considered δ=90 and e∈{Surgery, Device implant, Antibiotic, Kidney dialysis}. For the d-days ahead model, the feature is defined by considering δ+d as the parameter in the aforementioned definition, instead of δ.

5. Department-based features: we constructed the following features associated with room stays:

- ICU: this is a Boolean value that indicates whether a patient is admitted to an ICU.
- Emergency department (ED): this is a Boolean value that indicates whether a patient is admitted to the ED.

As in the aforementioned features, for the d-days ahead model, the feature is defined as 1 if the admission to ICU or ED happened before t-d, where *t* is the MRSA test time.

6. PHARMCLASS_k: there are 10 PHARMCLASS (penicillins, miscellaneous anti-infectives, cephalosporins, etc) in the data set. Each PHARMCLASS contains a list of antibiotics. For a patient, PHARMCLASS_k contains the number of antibiotic days from the MRSA testing date in the last 90 days. For the d-days ahead model, the feature is the number of antibiotic days in the 90 days before t-d.

7. Test duration days: for a patient p with an MRSA testing date t, we defined this feature as t-d-t’, if there exists a time t’, t(t’<t) at which an MRSA test was performed for p; otherwise, we defined this feature as 0.

### Machine Learning Classifiers

#### Overview

We explored the following machine learning methods: (1) logistic regression (LR; penalized) [17], (2) support vector machine [18], (3) random forest [19], (4) gradient-boosted classifiers, and (5) XGBoost. These methods have been used extensively on EHR data, and our goal was to understand which ones do well for the MRSA risk-prediction problems we considered in this study. We have described these methods in Multimedia Appendix 2 [17-19]. We also considered these methods with products of features, that is, of the form x_i_(t)•x_j_(t) where x_i_(t) and x_j_(t) are different components of the feature vector x(t). We also discuss the Shapley Additive Explanations (SHAP) technique for understanding feature importance in each model.

#### Model Explainability Using SHAP

**Figure 2:**
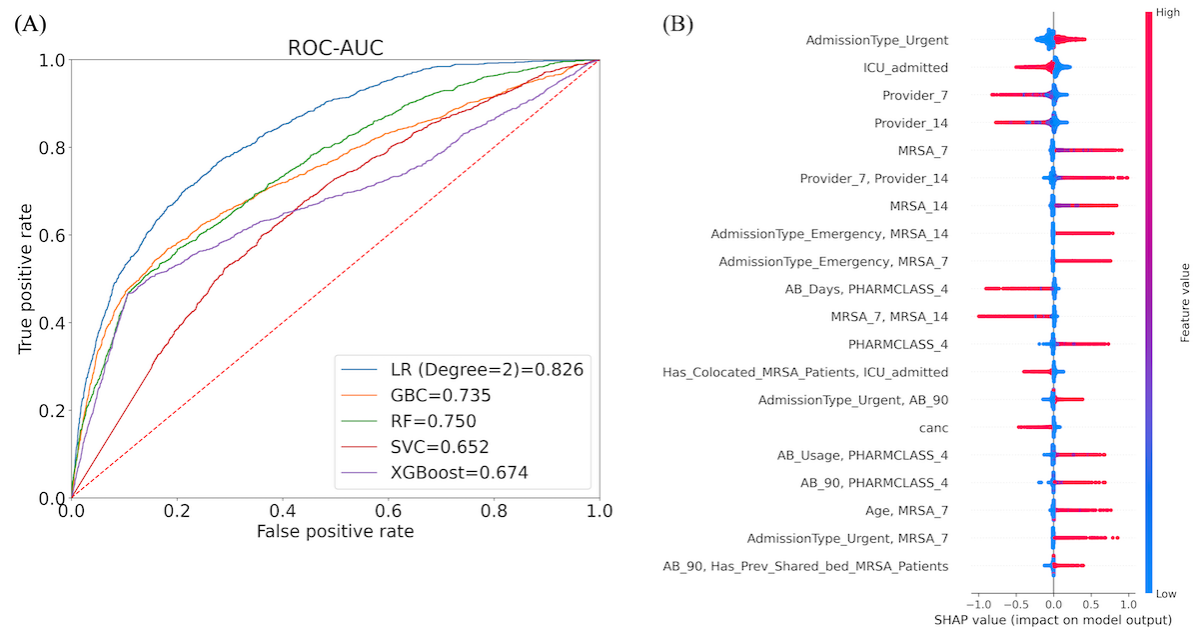
(A) Performance of models on the test data set: performance of different machine learning models on the entire University of Virginia data set. The penalized logistic regression (LR) model with degree-2 features performs best (the receiver operating characteristics-area under the curve [ROC-AUC] for the LR model without feature transformation to degree-2 is 0.734). (B) The most significant features in this model were identified using Shapley Additive Explanations (SHAP). GBC: gradient boosted classifier; RF: random forest; SVC: support vector classifier.

SHAP [20] is a visual feature-attribution process that has many applications in explainable artificial intelligence. It uses a game-theoretic methodology to measure the influence of each feature on the target variable of a machine learning model. Visual representations such as the one in Figure 2, referred to as a summary plot, are used to show the importance of features. The interpretations of this plot are as follows:

- The y-axis specifies the important features arranged from top to bottom regarding their importance (in descending order) to the response variable (the MRSA test result).
- The x-axis indicates the SHAP value of the corresponding feature. The SHAP value of a feature indicates the change in log odds that can be used to extract the probability of success. The color bar on the right-hand side indicates the gradient of log odds from low to high, with the color spectrum from blue to red.
- Each point in the SHAP plot for a feature represents an observation of the original data set.

#### Heterogeneous Risk-Prediction Models for Selected Subpopulations

To improve performance, we developed heterogeneous subpopulation-specific models as described in the subsequent sections.

##### Based on Testing History

Let K_p,t_∈{+1,-1} denote an MRSA test result for a patient *p* at time *t* in a hospital encounter. The testing history H_p,t_ is defined as H^j^_p,t_={K_p,ti_:1≤i≤j, t_j_<t_j-1_<...<t_1_<t}. No testing history exists for a newly admitted patient, expressed as H_p,t_=ø. The testing history, considering only the last test result, is expressed as H^1^_p,t_={K_p,t1_}. Similarly, the testing history, considering the last 2 test results, is expressed as H^2^_p,t_={K_p,t2_}. The number of patients with longer histories drops significantly; therefore, we limited our experiments to the last 2 test results. Table 1 presents the distribution of data points for the different subpopulations.

**Table 1:** Total number of observations and percentages of positive observations for the subpopulations based on different testing histories.

Previous test history	Total observations	Current test result (−1)	Current test result (+1)	Positive observations
None	27,612	24,371	3241	11.74
–1	11,338	10,179	1159	10.22
+1	3409	863	2546	74.68
(–1, –1)	4755	4320	435	9.15
(–1, +1)	635	198	437	68.82
(+1, –1)	480	328	152	31.67
(+1, +1)	1486	296	1190	80.00

##### Based on the Admission Source

Recall the Boolean feature named “From health care facility”, which is 1 if the admission source of a patient is a health care facility. We constructed 2 subpopulations based on whether this feature is 0 or 1; the distributions of these subpopulations and the percentage of positive observations in each are presented in Table 2.

**Table 2:** Total number of observations and percentages of positive observations for the subpopulations based on different categories.

Subpopulations		Total observations	Test result (*−*1)	Test result (+1)	Positive observations (%)
**Admission source**					
	Health care facility	2241	1619	622	27.76
	Other	42,840	36,198	6642	15.50
**Department**					
	ICU^a^	27,616	24,436	3180	11.52
	ED^b^	2538	1658	880	34.67
	Other	15,201	11,918	3283	21.60
**Hospital stays (days)**					
	≤15	39,221	32,541	6680	20.53
	>15	1643	1413	230	16.28
**Antibiotic use (days)**					
	≤90	30,776	25,065	5711	18.56
	>90	16,646	12,997	3649	21.92
	0	7097	6368	729	10.27
**Age group (years)**					
	0-50	14,269	12,093	2176	15.25
	≥50	27,638	23,008	4630	16.75

^a^ICU: intensive care unit.

^b^ED: emergency department.

##### Based on Department

Recall that both ICU and ED are 2 department-based features, which indicate whether the patient is in the ICU and ED, respectively. The distributions of the subpopulations and the percentage of positive observations are presented in Table 2.

##### Based on Hospital Stay

The feature “*Length of stay*” captures the number of days a patient has been in the hospital till time t-d, where t is the MRSA test date and d ≥ 0 is the parameter for the d-days ahead model. On the basis of this feature, we constructed 2 subpopulations. The first is the group of patients who have stayed in the hospital for at most 15 days, and the second is the group of patients who have stayed there for >15 days. The distribution of these subpopulations and the percentage of positive observations are presented in Table 2.

##### Based on Antibiotic Use

Three subpopulations were created based on the number of days for which a patient takes an antibiotic: (1) patients who never took any antibiotics, (2) patients who took antibiotics within the last 90 days from the MRSA testing date, and (3) patients who took antibiotics for more than 90 days from the MRSA testing date. The distribution of these subpopulations and the percentage of positive observations are presented in Table 2.

##### Based on Age Group

A total of 2 age group–specific patient subgroups, namely 0 to 50 and ≥50 years, are considered for the analysis. The distribution of these subpopulations and the percentage of positive observations are presented in Table 2.

##### Hierarchical Subpopulation-Based Models

**Figure 3:**
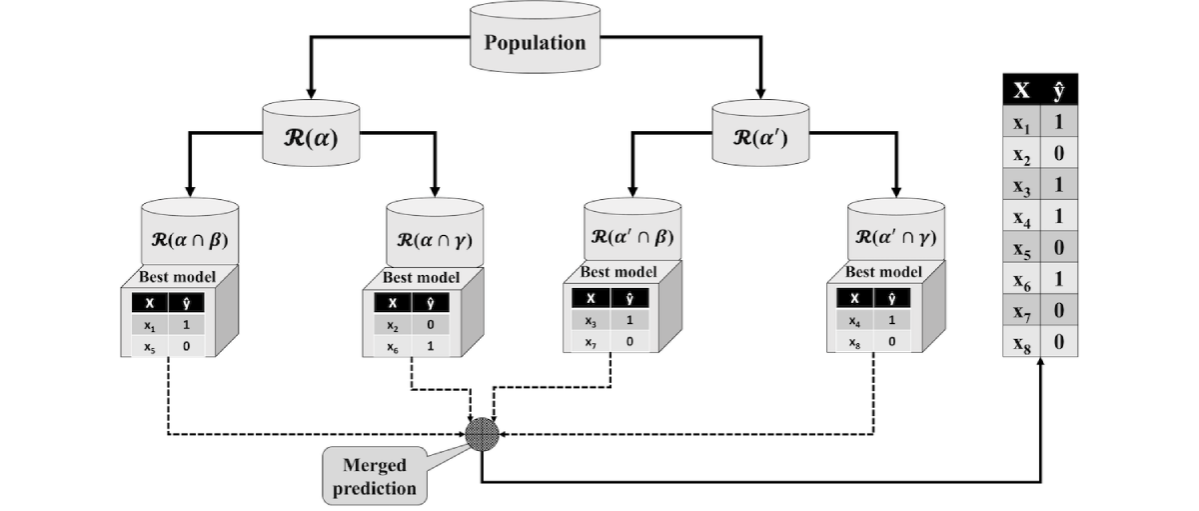
A schematic view of the hierarchical model architecture. In the figure, Xi represents the i-th observation, y is the model prediction, α is the patient subpopulation who are 0 to 50 years old, α' is the patient subpopulation who are more than 50 years old, β is the patient subpopulation who admitted to intensive care unit (ICU) department, γ is the subpopulation who admitted to the emergency department (ED), and R is a feature-based rule to aggregate data. For instance, R(α∩β) is a 0 to 50 age group patient subpopulation admitted to ICU. At level 1, the overall population is subdivided into two subpopulations based on the feature named “Age-group.” The patient subpopulation of age group (0 to 50 years) is mutually exclusive to the patient subpopulation of age group (>50 years). Each age group–specific subpopulation is further subdivided into the next level (level 2) based on another feature named “Department.” The patient subpopulation of the ICU department is mutually exclusive to the ED subpopulation. The training data are split based on the 2-level rules, and each patient subpopulation is trained using the best-fitted model. During the testing phase, each data point passes to the appropriate model using the same 2-level rules, and the best-fitted model predicts the outcome. The outcomes of all the models are merged back into the resultant prediction of this hierarchical model.

Figure 3 shows the schematic architecture of the hierarchical model. The construction steps of the hierarchical model are as follows:

- S1: we defined a set of feature-based rules R at each level to create mutually exclusive subpopulations:
  - At level 1, the rules on the feature named ‘Age-group’ are (1) R(α)=patient subgroup of 0 to 50 years old and (2) R(α’)=patient subgroup of more than 50 years old. Each rule creates a patient subpopulation. The patients in these two subpopulations are mutually exclusive, which can be expressed as: P(α)∩P(α’)=∅
  - At level 2, each age-group-specific subpopulation is subdivided based on another feature named “Department”. The rules on the ‘Department’ feature are (1) R(β)=patient subgroup of ICU and (2) R(γ)=patient subgroup of ED. Patients admitted to other departments are not considered in this model.
  - The two-level hierarchical structure creates a set of composite rules (combining rules of each level) at the leaf level that we call two-level rules. The rules are as follows: (a) R(α∩β), (b) R(α∩γ), (c) R(α’∩β), and (d) R(α’∩γ).
- S2: the training population is split based on the 2-level rules. Each training subpopulation is trained on several machine learning models, and the best-performing model is used for prediction.
- S3: each test observation is passed to the corresponding model using the 2-level rule. The observation with prediction is stored in a buffer. After completing all the testing observations, the buffer is treated as the model’s output.

#### Data Set for d-Days Ahead Prediction

We prepared a data set to observe the change of prediction performance to the change of *d*, which is discussed in the Methods section. For each *d*∈{1,2,…,7}, we created a data set, where the feature vector for a patient is generated based on the history of that patient till date *t-d*, where t is the MRSA testing date for that patient.

### Ethical Considerations

The data used in the paper was obtained through institutional review board approval and is fully anonymized. Therefore, there are no ethical considerations.

## Results

### Prediction Model for the Entire Population

We applied multiple machine learning models, including penalized LR, gradient-boosted classifier, Random Forest, support vector classifier, and XGBoost classifier (Multimedia Appendix 2), to the UVA Hospital MRSA patient data sets. We used an 80% to 20% split to construct the train and test data sets. Figure 2A shows the performance of the models. A model’s best set of hyperparameters was computed from the training data set using grid search and 10-fold cross-validation. Penalized LR was the best-performing model with the corresponding performance metrics: (1) the FNR score is 0.074, and (2) the ROC-AUC score is 0.826. Table 3 presents other performance metrics for this data set.

Given the same hyperparameter settings for the penalized LR model, the model performance (ROC-AUC) dropped to 0.734 when we did not consider the product features; therefore, this feature transformation provides a significant benefit. Using the SHAP technique discussed in the Methods section, we extracted the following key features from Figure 2B:

1. “AdmissionType_Urgent,” “ICU admitted,” “Provider 7,” and “Provider 14” are the top 4 features. Recall that “AdmissionType_Urgent” is a Boolean variable where the value 1 indicates the patient admitted as “Urgent.” Patients admitted as urgent have a higher likelihood of MRSA infection prediction. Similarly, “ICU admitted” is a Boolean feature where the value 1 indicates that the corresponding patient is admitted to the ICU department and is more likely to predict MRSA infection. On the other hand, “Provider 7” and “Provider 14” indicate the total number of providers a patient contacted in the last 7 and 14 days from the testing date. The higher value of these features is associated with high and negative values for the target feature (MRSA test). A high value comes from the rightmost color bar, and a negative value comes from the x-axis.
2. A high value of “MRSA 7” (which indicates the total number of patients with an MRSA-positive result a patient contacted in the last 7 days from the testing date) is associated with a high and positive value of the target feature (the MRSA test); this holds similarly for the “MRSA 14” feature.
3. In addition to single features, composite features also correlate more with MRSA infection prediction. For instance, “AdmissionType Emergency” and “MRSA 7” together (similar to “AdmissionType Emergency” and “MRSA 14”) are associated with high and positive values of the target feature (the MRSA test).
4. “PHARMCLASS_4” appears to be an important feature compared to the other PHARMCLASS features. In most cases, this variable is associated with high and positive values for the target feature.

The computational complexity of SHAP increases with the size of the test data set. The best-fitted model is passed to the SHAP explainer method, and it took 5 hours to generate the summary plot (Figure 2B) when the test data set contains 8174 observations and 4656 features. For the same best-fitted model, the SHAP explainer required 1 hour to generate the summary plot when the test data set contained the same number of observations, but the number of features was reduced to 97. Finally, the time was the same when the number of observations in the test data set was reduced to 817, and the number of features was 4656.

**Table 3:** Performance metrics of the best-performing model for each patient subpopulation based on room allocation, admission source, hospital stay, and antibiotic medication period.

Subpopulation	Model^a^	ROC-AUC^b^	AUPRC^c^	Sensitivity	Specificity	Precision	FPR^d^ or fallout	FNR^e^	*F*_1_-score	MCC^f^ score
Overall	LR^g^	0.826	0.504	0.684	0.797	0.406	0.203	0.074	0.510	0.400
ICU^h^	LR	0.876	0.428	0.775	0.826	0.381	0.174	0.036	0.511	0.455
ED^i^	LR	*0.936* ^j^	*0.882*	*0.878*	*0.886*	*0.800*	*0.114*	0.067	*0.837*	*0.749*
Other rooms	LR	0.752	0.451	0.574	0.793	0.389	0.207	0.110	0.463	0.320
From HCF^k^	LR	0.804	0.585	0.536	0.861	0.571	0.139	0.157	0.553	0.405
Not from HCF	LR	0.831	0.492	0.699	0.801	0.413	0.199	0.070	0.519	0.414
Hospital stay ≤15 days	LR	0.837	0.518	0.722	0.789	0.415	0.211	0.068	0.527	0.421
Hospital stay *>*15 days	LR	0.729	0.494	0.596	0.803	0.360	0.197	0.086	0.449	0.331
Antibiotic ≤90 days	LR	0.826	0.525	0.681	0.807	0.434	0.193	0.079	0.530	0.416
Antibiotic >90 days	LR	0.841	0.566	0.697	0.809	0.496	0.191	0.092	0.580	0.453
No antibiotic use	LR	0.834	0.328	0.734	0.721	0.201	0.279	*0.034*	0.315	0.275
Age group (0-50 years)	LR	0.782	0.482	0.613	0.777	0.364	0.223	0.094	0.457	0.325
Age group (≥50 years)	LR	0.833	0.514	0.660	0.817	0.428	0.183	0.079	0.520	0.408
Hierarchical model^l^	HM	*0.883*	0.490	*0.807*	0.832	0.440	0.168	*0.037*	0.569	0.507

^a^This column specifies the best-performing model.

^b^ROC-AUC: receiver operating characteristics-area under the curve.

^c^AUPRC: area under the precision-recall curve.

^d^FPR: false positive rate.

^e^FNR: false negative rate.

^f^MCC: Matthews correlation coefficient.

^g^LR: penalized logistic regression.

^h^ICU: intensive care unit.

^i^ED: emergency department.

^j^The best value for each performance metric is italicized.

^k^HCF: health care facility.

^l^For “Hierarchical model” (last row), the highlighted metric (in italics) indicates comparatively better performance than most of the other subpopulations.

### Effect of the Imbalanced Data Set

We evaluated the performance achieved using the different sampling techniques discussed earlier. First, as in the study by Hartvigsen et al [8], we used a random selection-based down-sampling technique to select majority-class observations and balance the number of observations between the majority and minority classes. The balanced data are split into train and test data. The ROC-AUC score of the best-performing model on the test data is 0.731. We used the synthetic minority oversampling technique (SMOTE) [21] on our data set to balance both majority and minority classes. The ROC-AUC score of the best-performing model on the test data is 0.896. Similar to the study by Hirano et al [9], we used SMOTE to balance the majority and minority classes in the imbalanced train and test data. The ROC-AUC score of the best-performing model on the test data is 0.903. However, when we evaluated the performance of the abovementioned models on a random test data set, the ROC-AUC score was significantly lower at 0.701. Thus, for our problem, the biased sampling techniques did not improve performance.

### Subpopulation-Specific Results

Our models and feature engineering cannot improve the ROC-AUC of 0.826. We now discuss the results of subpopulation-specific models.

#### Testing History–Based Analysis

**Figure 4:**
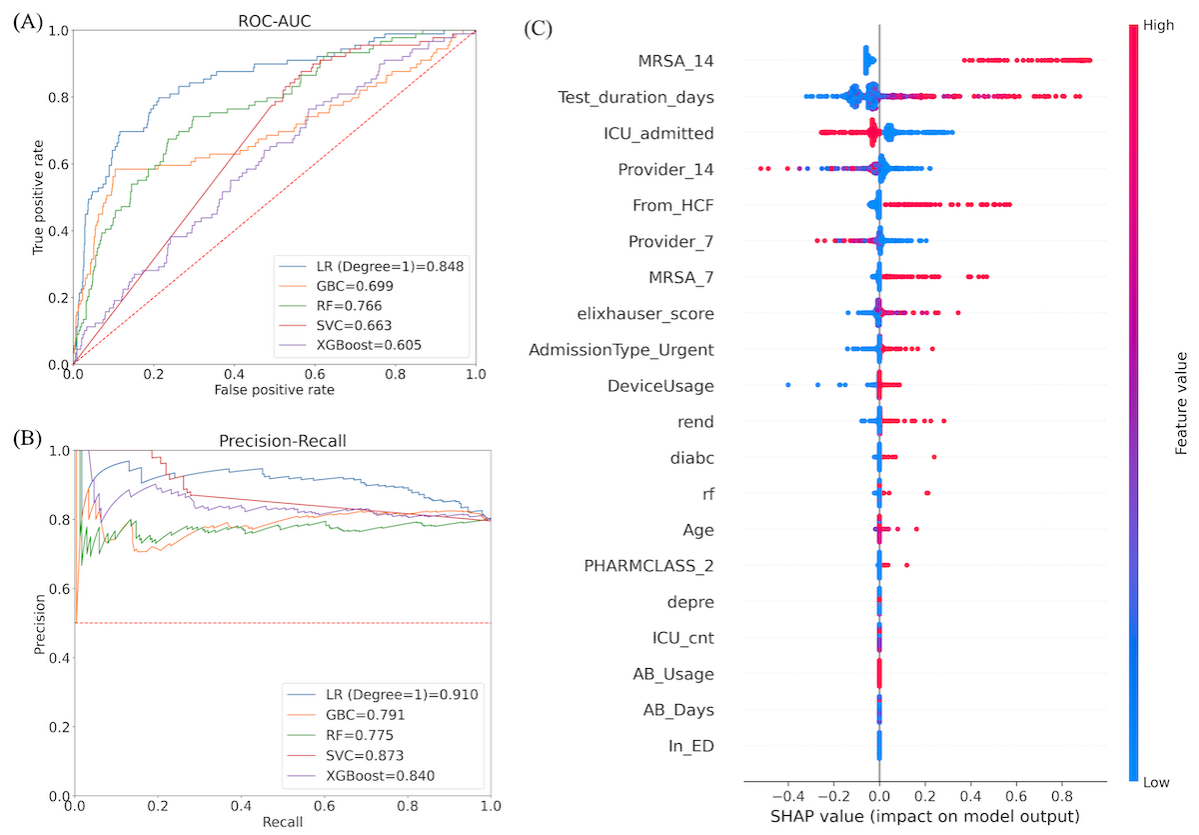
Results for best-performing subpopulations based on testing history: (A) Performance (receiver operating characteristics-area under the curve [ROC-AUC]) of different machine learning models for testing history (−1, −1), that is, the last 2 testing results are negative—penalized logistic regression (LR) has the best performance. (B) Performance (area under the precision-recall curve [AUPRC]) of different machine learning models for testing history (+1, +1), that is, the last 2 testing results are positive—penalized LR has the best performance. (C) Top features for (−1, −1) testing history–based subpopulation using the LR model. GBC: gradient boosted classifier; RF: random forest; SVC: support vector classifier.

The best-fitted model on testing history–based subpopulations (Table 4) showed the best performance on three subpopulations: (1) patients with a (−1) testing history: the best-fitted model had an ROC-AUC of 0.802; (2) patients with a (−1, −1) testing history: the best-fitted model had ROC-AUC of 0.848 and FNR of 0.035; (3) patients with a (+1, +1) testing history: the best model, in terms of the area under the precision-recall curve (AUPRC; Qi et al [22] suggested this metric for imbalanced data) performance metric, had an AUPRC of 0.910 (Figure 4B). The results for the other testing history–based data sets are shown in Multimedia Appendix 3.

Figure 4C shows the significant features (using the SHAP technique) for the (−1, −1) testing history–based subpopulations. The topmost feature (“MRSA 14”) is a network-based feature. Moreover, the network-based features are among the top 10 features. Among these features, “MRSA 7” and “MRSA 14” are positively associated with MRSA infection. In addition to the network features, the interval between the 2 MRSA tests is also important. In addition, patient comorbidity conditions have a significant correlation with MRSA infection.

**Table 4:** Performance metrics for the best-performing model for each patient subpopulation based on testing history.

Testing history	Model^a^	ROC-AUC^b^	AUPRC^c^	Sensitivity	Specificity	Precision	FPR^d^ or fall out	FNR^e^	*F*_1_-score	MCC^f^ score
None	LR^g^	0.814	0.406	0.689	0.749	0.276	0.251	0.054	0.394	0.311
(*−*1)	GB^h^	0.802	0.331	0.281	*0.953* ^i^	0.400	*0.047*	0.078	0.330	0.274
(+1)	LR	0.718	0.884	0.649	0.651	0.847	0.349	0.615	*0.735*	0.264
(−1,−1)	LR	*0.848*	0.402	0.697	0.855	0.332	0.145	*0.035*	0.449	*0.404*
(*−*1*,* +1)	SV^j^	0.613	0.781	0.295	0.897	0.867	0.103	0.639	0.441	0.209
(+1*, −*1)	SV	0.558	0.614	*0.875*	0.031	0.311	0.969	0.667	0.459	0.183
(+1*,* +1)	LR	0.761	*0.910*	0.595	0.787	*0.916*	0.213	0.667	0.721	0.308

^a^The “Model” column specifies the best-performing model (LR=penalized logistic regression classifier, GB=gradient boosting, and SV=support vector).

^b^ROC-AUC: receiver operating characteristics-area under the curve.

^c^AUPRC: area under the precision-recall curve.

^d^FPR: false positive rate.

^e^FNR: false negative rate.

^f^MCC: Matthews correlation coefficient.

^g^LR: logistic regression.

^h^GB: gradient boosting.

^i^The best value for each performance metric is italicized.

^j^SV: support vector.

#### Analysis for ICU and ED Subpopulations

**Figure 5:**
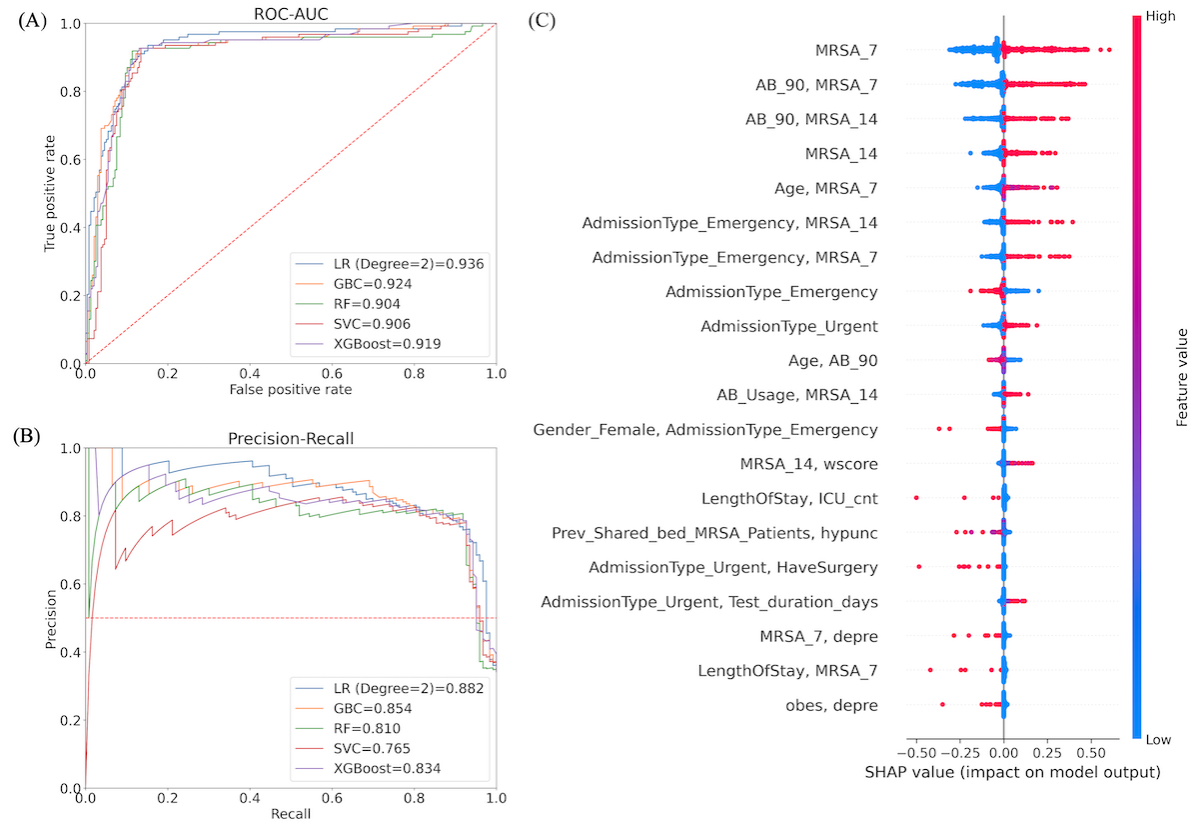
Results for the emergency department (ED) subpopulation that shows the best performance: (A) performance (receiver operating characteristics-area under the curve [ROC-AUC]) of different machine learning models—penalized logistic regression (LR) has the best performance. (B) Performance (area under the precision-recall curve [AUPRC]) of different machine learning models—penalized LR has the best performance. (C) Top features of the LR model. GBC: gradient boosted classifier; RF: random forest; SHAP: Shapley Additive Explanations; SVC: support vector classifier.

**Figure 6:**
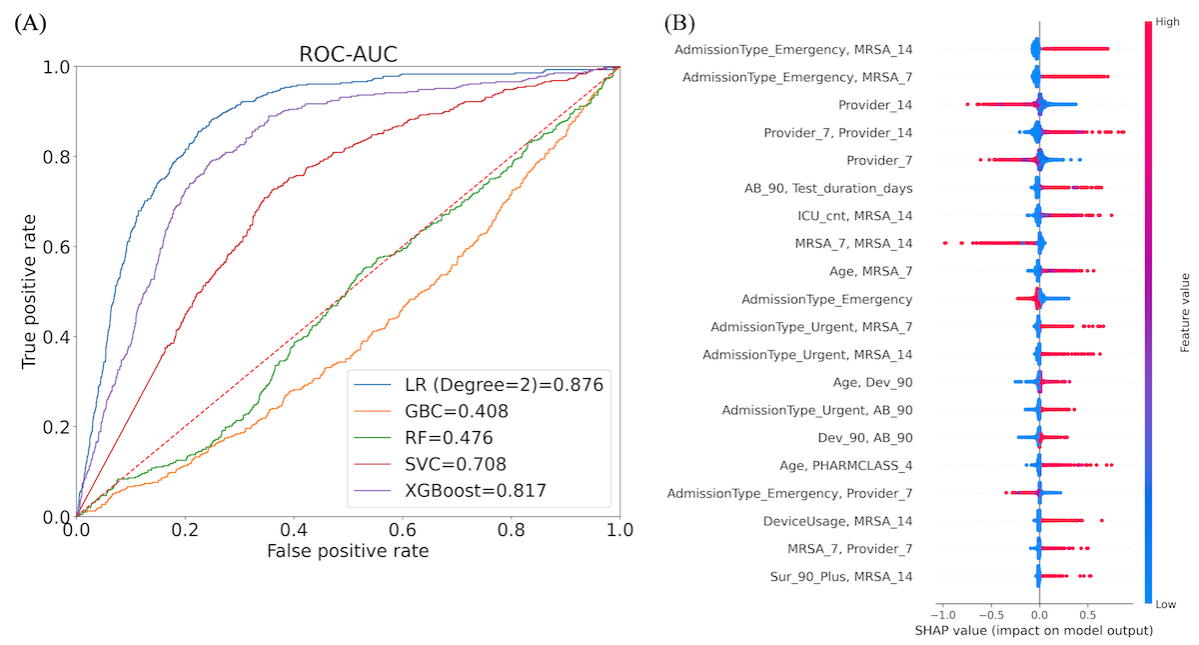
(A) Performance of different machine learning models for the intensive care unit subpopulation; the penalized logistic regression (LR) model performs best. (B) Top features of the LR model. GBC: gradient boosted classifier; RF: random forest; SHAP: Shapley Additive Explanations; SVC: support vector classifier.

We developed models for other subpopulations, and the performance of the best-fitted models for these subpopulations is reported in Table 3. We found that the best performance is for the ED subpopulation in terms of both ROC-AUC and AUPRC. The ROC-AUC value for the best-fitted model is 0.936 (Figure 5A), and the AUPRC value for the best-fitted model is 0.882 (Figure 5B). Regarding the FNR, the model best performs for the subpopulation without antibiotics. The FNR score obtained using the best-performing model for this data set is 0.034. The subpopulation with the second-best performance is the ICU subpopulation (Figure 6), and the corresponding FNR score is 0.036. The results for the other subpopulations are presented in Multimedia Appendix 4.

Figure 6B shows the significant features (using the SHAP technique) of the best model for the ICU subpopulation. The top 5 network-based features and the frequency of network features in the top 20 again demonstrate the significance of the network structure. Some of the nonnetwork features that appear to be important are the patient’s age, use of antibiotics in the last 90 days, use of a device in the last 90 days, test duration days, PHARMCLASS 4, and emergency and urgent-type patient admission.

Figure 5C shows the significant features (using the SHAP technique) for the best-performing model for the ED subpopulation. The top 7 features have network features. The top influential feature for the ICU subpopulation is “MRSA 14,” whereas the top significant feature for the ED subpopulation is “MRSA 7.” Unlike in the ICU, the patient’s gender, length of stay, and comorbidity conditions are also crucial in addition to network features.

#### Hierarchical Models

The performance of this model is presented in Table 3. This model’s ROC-AUC and FNR scores are 0.883 and 0.037, respectively. This model performs better than most subpopulation-based models except for the ED subpopulation-based models.

### Importance of Network Features

The best-fitted model performance on the entire data set shows the best performance (Table 3) regarding ROC-AUC and FNR when we use network features. The corresponding ROC-AUC score is 0.826, and the FNR score is 0.074. Without the network features, the ROC-AUC score for the best-fitted model is 0.714, and the FNR score is 0.107 (Table 5).

The ROC-AUC score improved by approximately 16%, and the FNR score improved by approximately 31% because of the network features. The influence of network features is also significant in the models for the ICU and ED patient subpopulations. The performance metric ROC-AUC improved by approximately 27% for the ICU department patient subpopulation, and the FNR score improved by approximately 58%. For ED patient subpopulations, the performance metric ROC-AUC improved by approximately 30%, the FNR score improved by approximately 69%, and the AUPRC score improved by approximately 50%.

Network features also improve the performance of the best-fitted model for testing history–based subpopulations (Tables 3 and 6).

The ROC-AUC performance metrics for the best-fitted model (−1) testing the history-based subpopulation improved by approximately 11%. For (−1, −1) testing the history-based subpopulation, the best-fitted model performance improved by approximately 25% on the ROC-AUC score and approximately 35% on the FNR score.

**Table 5:** Performance metrics of the best-performing model for each patient subpopulation based on room allocation, admission source, hospital stay, and antibiotic medication period after excluding the network features.

Subpopulation	Model^a^	AUC^b^	AUPRC^c^	Sensitivity	Specificity	Precision	Fall out	FNR^d^	*F*_1_-score	MCC^e^ score
Overall	LR^f^	0.714	0.383	0.610	0.709	0.314	0.291	0.107	0.415	0.257
ICU^g^	LR	0.690	0.311	0.547	0.760	0.262	0.240	0.085	0.354	0.233
ED^h^	LR	0.722	0.589	0.593	0.705	0.496	0.295	0.220	0.541	0.287
Other rooms	LR	0.692	0.346	0.631	0.672	0.308	0.328	0.113	0.414	0.243
From HCF^i^	LR	0.594	0.340	0.348	0.799	0.375	0.201	0.220	0.361	0.151
Not from HCF	LR	0.721	0.367	0.631	0.704	0.298	0.296	0.095	0.405	0.261
Hospital stay ≤15 days	LR	0.718	0.381	0.615	0.712	0.311	0.288	0.103	0.413	0.261
Hospital stay >15 days	LR	0.595	0.262	0.615	0.566	0.209	0.434	0.112	0.312	0.133
Antibiotic ≤90 days	LR	0.732	0.402	0.634	0.721	0.336	0.279	0.101	0.439	0.288
Antibiotic >90 days	LR	0.707	0.434	0.621	0.683	0.361	0.317	0.138	0.457	0.261
No antibiotic use	LR	0.661	0.236	0.520	0.696	0.178	0.304	*0.080* ^j^	0.265	0.145
Age group (0-50 years)	LR	0.715	0.404	0.617	0.703	0.298	0.297	0.100	0.402	0.251
Age group (≥50 years)	LR	0.721	0.357	0.628	0.714	0.295	0.286	0.090	0.401	0.265

^a^The “Model” column specifies the best-performing model (LR=penalized logistic regression classifier).

^b^AUC: area under the curve.

^c^AUPRC: area under the precision-recall curve.

^d^FNR: false negative rate.

^e^MCC: Matthews correlation coefficient.

^f^LR: logistic regression.

^g^ICU: intensive care unit.

^h^ED: emergency department.

^i^HCF: health care facility.

^j^italics.

**Table 6:** Performance metrics for the best-performing model for each patient subpopulation based on testing history after excluding the network features.

Testing history	Model^a^	AUC^b^	AUPRC^c^	Sensitivity	Specificity	Precision	Fall out	FNR^d^	*F*_1_-score	MCC^e^ score
None	LR^f^	0.660	0.221	0.565	0.660	0.187	0.340	0.084	0.281	0.153
(−1)	GB^g^	0.723	0.233	0.031	0.996	0.467	0.004	0.098	0.058	0.099
(+1)	LR	0.685	0.851	0.623	0.628	0.821	0.372	0.620	0.708	0.224
(−1, −1)	LR	0.677	0.196	0.663	0.615	0.151	0.385	0.054	0.246	0.164
(−1, +1)	SV^h^	0.637	0.797	0.625	0.615	0.786	0.385	0.579	0.696	0.223
(+1, −1)	SV	0.507	0.356	0.375	0.656	0.353	0.344	0.323	0.364	0.031
(+1, +1)	LR	0.691	0.881	0.605	0.719	0.887	0.281	0.667	0.719	0.267

^a^The “Model” column specifies the best-performing model (LR=penalized logistic regression, GB=gradient boosting, and SV=support vector).

^b^AUC: area under the curve.

^c^AUPRC: area under the precision-recall curve.

^d^FNR: false negative rate.

^e^MCC: Matthews correlation coefficient.

^f^LR: logistic regression.

^g^GB: gradient boosting.

^h^SV: support vector.

### d-Days Ahead Model Prediction

**Figure 7:**
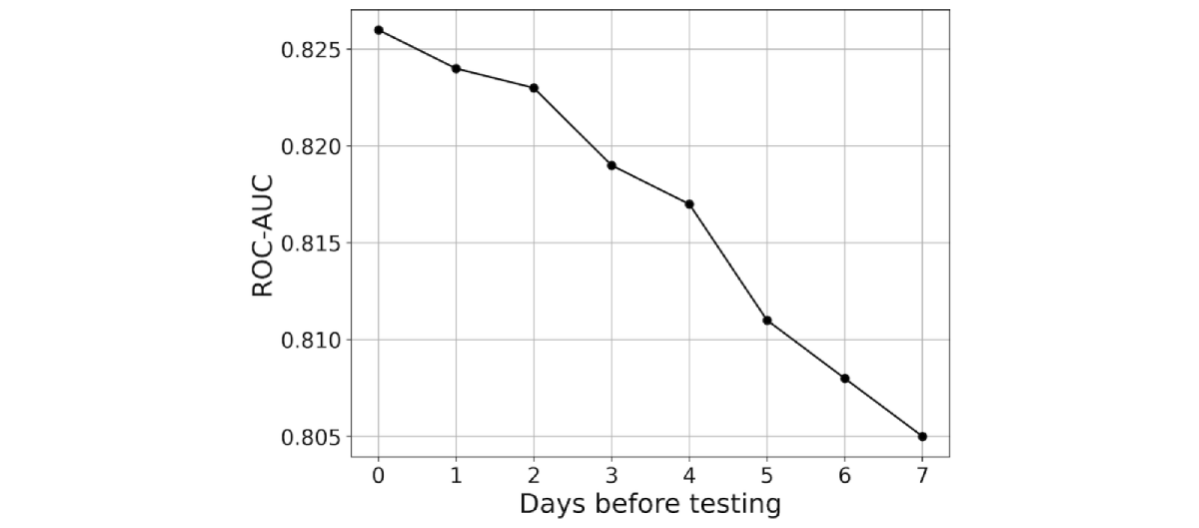
d-days ahead prediction: performance (receiver operating characteristics-area under the curve [ROC-AUC]) of best model versus d. The performance drops gradually with d.

We now examine how well the test results can be predicted per the *d*-days ahead model. We expected the performance to drop as *d* increases, as shown in Figure 7, which shows the ROC-AUC score of the best-fitted model (for the data set corresponding to *d*-days before the test, as described in the Methods section) versus *d*. Note that the performance decays significantly with *d*.

## Discussion

### Principal Findings

Our results demonstrate that clinically relevant models can be developed for predicting MRSA test results with high accuracy using a combination of clinical and nonclinical features from EHR data. In particular, features of contact networks (eg, “MRSA 7,” “MRSA 14,” “Provider 7,” and “Provider 14”) constructed from EHR data are quite significant in our models. Tables 5 and 6 show the performance of the models on the same group of data sets without considering the network features. The empirical results establish that the network features have a significant impact (model performance ROC-AUC improves by > 15%) on MRSA infection prediction.

We took the simplest approach to network construction, which views edges as unweighted, and did not consider heterogeneity in contacts, for example, based on types of providers. It is interesting that even the simplest approach improves performance. While more characteristics of networks and edge weights could be considered and these might improve the performance, the value of our simple approach is that it is easier to construct and is likely more generalizable and robust because there might be uncertainties in some of these additional characteristics.

In addition to network features, we observed that features associated with antibiotic use (“Antibiotic days”, “Antibiotic days in last 90 days”, “Antibiotic days in last 90+ days”, “PHARMCLASS_1” to “PHARMCLASS_10”, etc.), different kinds of events in the past 90 days (eg, kidney dialysis, device use, and any surgery), and comorbidity conditions such as diabetes without complications (diab or diabunc), hypothyroidism (hypothy), uncomplicated hypertension (hypunc), the Charlson score, the Elixhauser score, the weighted version of the Elixhauser score using the van Walraven algorithm (wscore vw), the weighted version of the Elixhauser score using the *Agency for Healthcare Research and Quality* (AHRQ) algorithm (wscore ahrq), and the weighted version of the Charlson score (wscore) are also predictive; many of these have been identified as important in prior work.

The penalized LR model with degree-2 polynomial features performs best in almost all settings, using a new class of network-based features derived from EHR data. Our results also showed the utility of heterogeneous models for different subpopulations instead of just one model for the entire population. In particular, we obtained good performance for subpopulations in an ICU or ED and those with certain test histories. We also observed that the performance degrades gradually for a *d*-days ahead prediction.

The testing policy is fairly systematic for patients in the ICU. Therefore, we expect the model for ICU subpopulations to be quite robust and generalizable to data sets from other locations. On the other hand, it is important to note that testing in the entire patient population is generally not completely systematic and might have biases because it is administered per physician request. It is unclear what the impact of these biases would be on the model’s generalizability. A mitigating factor is that the model for the entire population is quite close to that for the ICU, and many of the significant factors are the same. This suggests that the model for the entire population might also be quite robust. Future studies on other data sets are required to determine the generalizability of these models.

Our prediction model for a patient on day t only used features that were available for that patient before day t. This included the network features. Therefore, if a patient was in the hospital for <7 days, the “MRSA 7” and “Provider 7” feature values will be 0, and if a patient was in the hospital for <14 days, the “MRSA 14” and “Provider 14” feature values will be 0. It is possible that the predictive model would be more informative for patients who have a longer history in the hospital, but even this is an important patient population from a clinical perspective.

Finally, we noted that the simple penalized LR model seems to work quite well when given more complex features, such as second-degree features. It is not completely clear why this works much better than the other methods, namely support vector machine, random forest, gradient-boosted classifiers, and XGBoost. One possible explanation can be because of the model parsimony of the penalized LR. Further research on model validation can be useful. One advantage of our analysis is that the penalized LR method is easy to interpret.

Our models are the most useful for clinical decisions about empiric antibiotic use. For instance, if the test prediction is negative, a clinician could be more comfortable starting an antibiotic treatment. If the test prediction is positive in the context of a newly identified infection, a clinician might consider the benefits of starting an anti-MRSA antibiotic. Isolation precautions are known to have many adverse effects (eg, fewer clinician visits to the room, patient depression, and noninfectious adverse events such as blood clots), although they help in reducing transmission. If the *d*-days ahead result is negative in a current patient with a positive MRSA result, an epidemiologist may adjust for an earlier test for clearance of isolation precautions.

### Comparison With Prior Work

Machine learning using EHR data for clinical informatics is a very active area of research [23,24]. Diverse kinds of statistical and machine learning methods, including deep-learning algorithms, have been used to predict important clinical events (eg, hypertension, diabetes, chronic obstructive pulmonary disease, arrhythmia, asthma, gastritis, dementia, delirium, *Clostridium difficile* infection, and HAIs) using EHR data [8,9,12,13,25-29]. In the context of HAIs, risk-prediction models have been developed for several MDROs. We have briefly discussed examples of such studies to illustrate the types of questions and methods that have been considered, with a focus on MRSA.

Hartvigsen et al [8] and Hirano et al [9] studied a similar problem, namely, predicting MRSA test outcomes, using the Medical Information Mart for Intensive Care III and IV data sets, respectively. These data sets are critical care data sets comprising 12 years (2001 to 2012 and 2008 to 2019, respectively) of patient records from the Beth Israel Deaconess Medical Center Intensive Care Unit in Boston, Massachusetts [11]. Hartvigsen et al [8] show high performance for the prediction of MRSA test outcomes 1 day ahead using subsampled data. Hirano et al [9] achieve high performance (an ROC-AUC value of 0.89) for a slightly different patient subpopulation using the SMOTE [21] technique for handling data imbalance. Rhodes et al [12] consider a slightly different question regarding MRSA infection 72 hours after admission. They show that the Classification Tree Analysis has good performance for the population of patients from the Northwestern Memorial Hospital and Lake Forest Hospital. A review by Tang et al [13] notes that penalized LR, decision tree, and random forest are the preferred methods for antimicrobial resistance prediction.

A significant challenge hern all MRSA risk-prediction problems (including our study) is that the data are quite imbalanced because the fraction of positive observations is quite small. Consequently, the performance of most machine learning methods can be affected. A common strategy to address this issue has been to construct data sets using different kinds of sampling techniques, including biased sampling [8,10] and SMOTE [30]. While this kind of approach can appear to have very good performance on a similarly constructed test data set, the true performance on an unbiased data set might be reduced (as discussed in the study by Pencina et al [31] and in our Results section), which impacts its performance when used in practice. According to the study by Soltanzadeh and Hashemzadeh [30], resolving the class distribution problem using synthetic or biased data constructed in this manner causes many issues such as (1) generalization problems because of noisy samples; (2) uninformative samples; and (3) newly created points being close to the minority class points, which often create points around the decision boundary. Azizi et al [32] and Kokosi and Harron [33] note that (1) the use of synthetic data in the decision-making process and (2) the problem of attribute disclosure are other limitations of using synthetic data.

Our study differs from prior work in 3 ways. First, we used network features in addition to other EHR-based features in our risk-prediction models. It has been shown that network properties are predictive of infection risk, for example, Klein et al [34] showed that patient degree is associated with vancomycin-resistant enterococci risk. Similarly, Riaz et al [35] show that local colonization pressure, which is based on the network structure, is associated with *C. difficile* infection (CDI) risk. Similarly, Miller et al [36] show that household exposure (which can also be viewed as a network effect) increases CDI risk. However, our work is the first to explicitly consider EHR-based features for MRSA test prediction as a machine learning task that can be used in a clinical setting. Second, we identified heterogeneous models for specific patient subgroups and showed that these have significantly better performance. Finally, we developed our prediction models without any biased sampling techniques.

### Limitations

We have not been able to improve the ROC-AUC performance of our models above 0.90. Data imbalance and patient diversity could be significant reasons for this performance. As noted earlier, MRSA infections are fairly rare, and for the problem of MRSA test results, only about 15% of the results are positive. We also note that there are many other notions of MRSA risk, such as the risk of severe outcomes and MRSA acquisition, which we study here. These notions are harder to formalize and learn because the data sets would become even more biased than what we consider here, and new methods are needed for them.

While our results show that network features are the most predictive, there might be uncertainties in inferring them from the EHR data. We note that these (eg, the #providers within a time interval) are not directly available in the patient’s EHR data; we are inferring them through colocation information. It is possible that many interactions are not recorded accurately or the times might not be accurate. More work is needed to fully understand the impact of these uncertainties.

Another issue is the testing bias. As discussed earlier, the entire patient population data set has biases because testing is not very systematic in general. This might have an impact on the model’s performance when applied to data sets from other hospitals, and the model would have to be retrained. However, the model structure and specific features might still be relevant, especially because they hold for the ICU patient subpopulation, for which testing is more systematic.

### Conclusions

Preprocessing by clustering has been useful in many applications. One challenge in using this approach is that a distance metric needs to be defined, which is difficult due to the diversity of features. For instance, some features are datetime related, some are Boolean and categorical, while others are real valued. A possible extension is to transform the features into a latent space, where distances can be computed. Additional feature engineering and more advanced machine learning methods might be useful for further improving performance. In particular, text analysis might be helpful in further improving the performance.

## Appendix

Multimedia Appendix 1 Machine learning model evaluation metrics.

Multimedia Appendix 2 Machine learning models.

Multimedia Appendix 3 Test history–based results and machine learning model hyperparameters.

Multimedia Appendix 4 Patient subpopulation-based results and machine learning model hyperparameters.

## Abbreviations

AUPRC: area under the precision-recall curve
ED: emergency department
EHR: electronic health record
FNR: false negative rate
HAI: health care–associated infection
ICU: intensive care unit
LR: logistic regression
MDRO: multidrug-resistant organism
MRSA: methicillin-resistant Staphylococcus aureus
ROC-AUC: receiver operating characteristics-area under the curve
SHAP: Shapley Additive Explanations
SMOTE: synthetic minority oversampling technique
UVA: University of Virginia
