# Supplementary material for: Improving Risk Prediction of Methicillin-Resistant Staphylococcus aureus Using Machine Learning Methods With Network Features: Retrospective Development Study
Source: JMIR AI. 2024 May 16;3:e48067. doi: 10.2196/48067 (PMC11140275; doi:10.2196/48067)
Supplement: Multimedia Appendix 1 [file ai_v3i1e48067_app1.pdf]

Multimedia Appendix 1

Model evaluation metrics

The performance metrics of a classification machine learning model are generated from a confusion matrix. Table 1 shows a schematic view of a confusion matrix where cells represent the following classification metrics:

- **True positive (TP):** the number of observations that are Positive in the original observation and predicted as Positive by the classifier.
- **True Negative (TN):** the number of observations that are Negative in the original observation and predicted as Negative by the classifier.
- **False positive (FP):** the number of observations that are Negative in the original observation but predicted as Positive by the classifier. It is also known as a Type I error.
- **False Negative (FN):** the number of observations that are Positive in the original observation but predicted as Negative by the classifier. It is also known as a Type II error.

Table 1: Confusion matrix

		**Prediction outcome**		
		$\hat{p}$	$\hat{n}$	**total**
**actual value**	**p**	True Positive (TP)	False Negative (FN)	P
	**n**	False Positive (FP)	True Negative (TN)	N
	**total**	$\hat{P}$	$\hat{N}$	

The following machine learning model evaluation metrics are derived from the above confusion matrix:

- **Precision or Positive predictive value:** The precision of a model is the ratio between the number of correct positive predictions and the overall number of positive predictions. The formula of this metric is as follows:

  $$\text{Precision} = \frac{TP}{TP+FP}$$

  Higher precision of a model indicates that the model has higher predictive power in both positive and negative observations.

- **Sensitivity or Recall or True positive rate:** Sensitivity is the ratio between the number of positive observations predicted correctly and the total number of positive observations. The formula is as follows:

  $$\text{Sensitivity}= \frac{TP}{TP+FN}$$

  The higher sensitivity of a model indicates that the model has a high capability to predict positive observations.

- **Specificity or True negative rate:** The specificity of a model indicates the ratio between the number of negative observations predicted correctly and the overall number of negative observations. The formula to compute this metric is as follows:

  $$\text{Specificity} = \frac{TN}{TN+FP}$$

  The higher specificity of a model indicates that the model has a high capability to predict negative observations.

- **Fall out or False positive rate:** Fall out of a model is the ratio between the number of incorrect positive predictions and the total number of negative observations. The formula to compute this metric is as follows:

  $$\text{False positive rate (FPR)} = \frac{FP}{FP+TN} = 1 - \text{Specificity}$$

- **False negative rate (FNR):** The false negative rate of a model is the ratio between incorrect negative prediction and overall positive observations. We changed the denominator to overall negative prediction observations. The metric indicates the incorrect negative observation prediction probability of the model. The formula to compute this metric is as follows:

  $$\text{False negative rate (FNR)} = \frac{FN}{FN+TN}$$

  Lower this value specifies that the model has a lower probability of misclassifying positive observation.

- **ROC-AUC:** An ROC curve (receiver operating characteristic curve) is a graphical representation of the performance of a classifier model at all classification thresholds. The graph is plotted based on two other metrics: (i) True positive rate (TPR) or recall and (ii) False positive rate (FPR).

  A ROC curve plots TPR vs. FPR at different classification thresholds. A change in the classification threshold will change the curve as well. For instance, lowering the classification threshold will classify more items as positive, which will increase both FP and TP.
  An AUC is an area under the ROC curve. One interpretation of AUC is the probability that the classifier ranks a random positive example more highly than a random negative example. The AUC values range from 0 to 1. An AUC = 0.0 means the prediction of the classifier is 100% wrong, an AUC = 1.0 means the prediction of the classifier is 100% correct, and an AUC = 0.5 indicates the prediction power of the model is the same as random selection.

- **AUPRC:** Like the ROC-AUC curve, the AUPRC curve computes both precision and recall values under all threshold probabilities. This metric uses the average precision score as a measure of the combined precision-recall score. A higher AUPRC score indicates that both precision and recall values are higher, and the model generates fewer false positive and fewer false negative values. The range values of the AUPRC score range from 0 to 1, where 0 indicates the True positive value is 0 and 1 indicates both False positive and False negative values are zeros.

- **F score:** The F score of a model is a harmonic mean of precision and recall. The formula of this metric is as follows:

$$F_\beta \text{score} = \frac{(1+\beta^2)\times Precision \times Recall}{\beta^2 \times Precision + Recall}$$

  The common values of β are 0.5, 1.0, or 2. We used β = 1 for our model. An F_1 score close to 1 indicates that the model is performing very well, whereas an F_1 score of less than 0.5 indicates a poor-performing model.

$$F_{0.5} \text{score} = \frac{1.25\times Precision \times Recall}{0.25\times Precision + Recall}$$

$$F_1 \text{score} = \frac{2\times Precision \times Recall}{Precision + Recall}$$

$$F_2 \text{score} = \frac{5\times Precision \times Recall}{4\times Precision + Recall}$$

- **Matthew's correlation coefficient (MCC) score:** MCC score is a correlation coefficient that is calculated using all four values in the confusion matrix. MCC score ranges from −1 to 1. A model with a score of +1 or close to +1 is perfect, and −1 or close to −1 is poor. The formulation of this metric is:

$$\text{MCC score} = \frac{TP\times TN - FP\times FN}{\sqrt{(TP+FP)+(TP+FN)+(TN+FP)+(TN+FN)}}$$
