# Supplementary material for: Improving Risk Prediction of Methicillin-Resistant Staphylococcus aureus Using Machine Learning Methods With Network Features: Retrospective Development Study
Source: JMIR AI. 2024 May 16;3:e48067. doi: 10.2196/48067 (PMC11140275; doi:10.2196/48067)
Supplement: Multimedia Appendix 2 [file ai_v3i1e48067_app2.pdf]

Machine learning classifiers

We explore the following machine learning methods for our experiments.

Penalized logistic regression

The Penalized logistic regression model is a binary classifier used to study the effect of predictor variables on categorical outcomes and is one of the most commonly used classifiers [21]. The penalized logistic model involves a function of the form $\frac{1}{1+\exp(-\omega^T x)}$, where ω is the weight vector, which is learned by optimizing the following loss function with L_1 regularization (penalty):

$$L(\omega;\beta;\lambda) = \lambda||\omega|| + \sum_{(x,y)\in S} \ln\left(1+\exp\left(-y(\omega^T x+\beta)\right)\right) \quad (1)$$

Support Vector Machine (SVM)

SVM is another popular method for binary classification, which uses the function $\mathrm{sign}(\omega^T x+\omega_0)$. The model is trained by optimizing the hinge loss function [8]

$$\lambda||\omega||^2 + \frac{1}{|S|}\sum_{(x,y)\in S} \max\{0, 1-y(\omega^T x+\omega_0)\} \quad (2)$$

However, SVM is computationally more expensive to train. In our experiments, it was difficult to train SVM with the product of features. Additionally, the performance of SVM was worse than other models, but is presented here because of its widespread use.

Tree-based ensemble methods: Random Forest, Gradient Boosted Decision Tree (GBDT), and XGBoost

The random forest classifier is the simplest ensemble method, in which each classifier is a decision tree trained on a random sample of the data, and each tree has a unit weight in the ensemble [4]. Therefore, random forests are computationally efficient and have low variance. The number of trees and the depth of the tree are two hyperparameters of a random forest classifier.

A Gradient-boosted decision tree is another tree-based ensemble method where several decision trees are used as weak learners, and together, all the weak learners form a strong and effective classifier. Initially, each sample is weighted equally, then the first sample is trained by the first weak classifier, and so on. After learning, we reduced the weight of correctly classified samples and increased the weight of mistaken samples. Residuals are computed from the mistaken samples, and a weak classifier is trained based on the previous weak classifier's residual error. In this way, GBDT can reach the classification target by decreasing the residual error in the training process. XGBoost classifier is an improved version of the GBDT classifier in terms of computing speed, generalization, and scalability.
