# Supplementary material for: Improving Risk Prediction of Methicillin-Resistant Staphylococcus aureus Using Machine Learning Methods With Network Features: Retrospective Development Study
Source: JMIR AI. 2024 May 16;3:e48067. doi: 10.2196/48067 (PMC11140275; doi:10.2196/48067)
Supplement: Multimedia Appendix 3 [file ai_v3i1e48067_app3.pdf]

Multimedia Appendix 3

Test history-based results

Figure 1: Performances of different machine learning models on different testing history-based patient subpopulations.

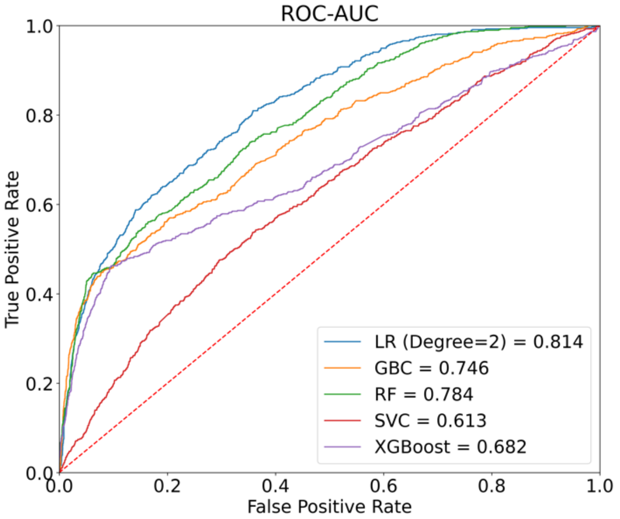

(a) Subpopulation of patients with no past testing history.

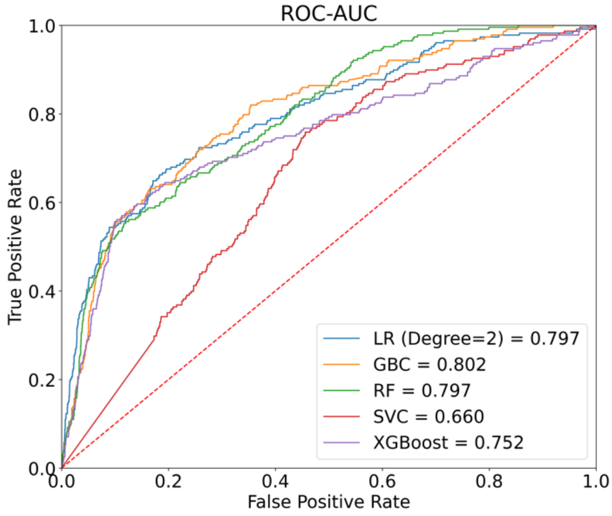

(b) Subpopulation of patients with (-1) test history.

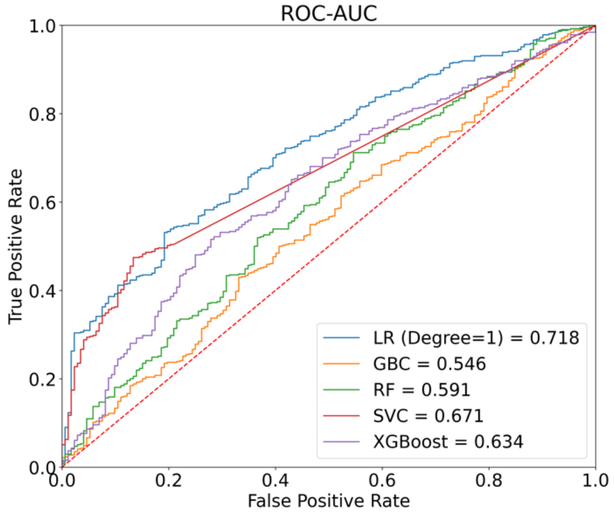

(c) Subpopulation of patients with (+1) test history.

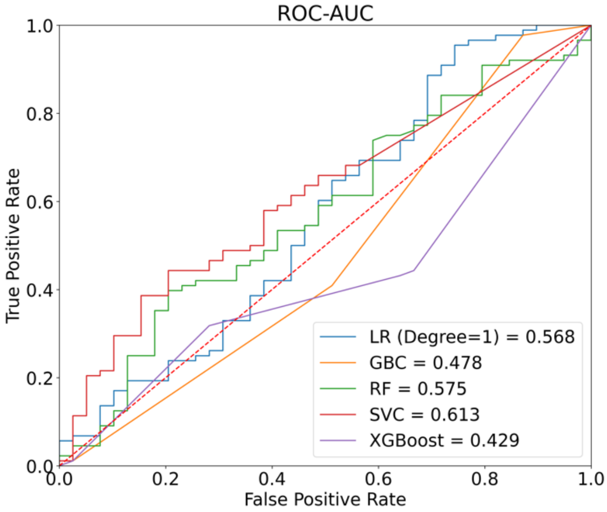

(d) Subpopulation of patients with (-1, +1) test history.

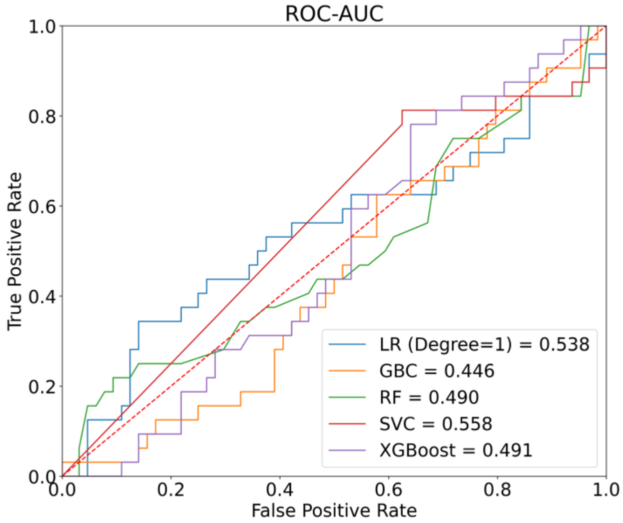

(e) Subpopulation of patients with (+1, -1) test history.

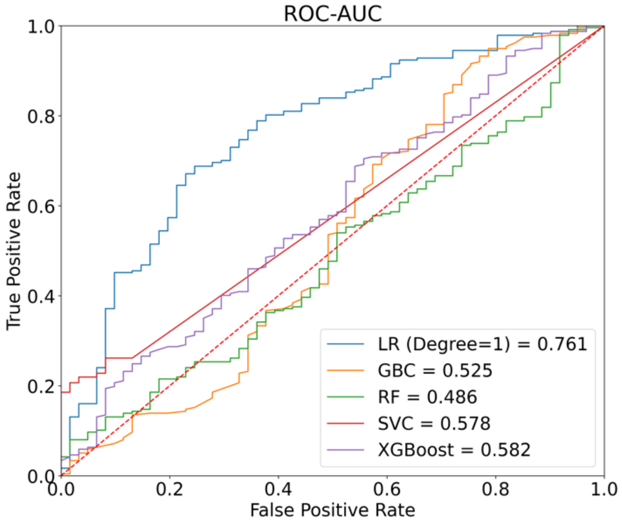

(f) Subpopulation of patients with (+1, +1) test history.

Model hyperparameters

Table 1: The hyperparameters of the best-performing model for the testing history-based subpopulation. The 'Model' column specifies the best-performing model. The abbreviations are LR=Penalized logistic regression classifier, RF=Random Forest, GB=Gradient boosted, and SV=Support vector.

**Testing history**	**Model**	**Hyperparameter**
None	LR	C=0.01, penalty=l_1, degree=2
(−1)	GB	Learning rate=0.1, maxdepth=2, no of estimators=200
(+1)	LR	C=0.1, penalty=l_1
(-1, -1)	LR	C=0.01, penalty=l_1
(-1, +1)	SV	C=1.0, kernel=poly, degree=1
(+1, -1)	SV	C=1.0, kernel=poly, degree=1
(+1, +1)	LR	C=0.01, penalty=l_2
