# Supplementary material for: Improving Risk Prediction of Methicillin-Resistant Staphylococcus aureus Using Machine Learning Methods With Network Features: Retrospective Development Study
Source: JMIR AI. 2024 May 16;3:e48067. doi: 10.2196/48067 (PMC11140275; doi:10.2196/48067)
Supplement: Multimedia Appendix 4 [file ai_v3i1e48067_app4.pdf]

Multimedia Appendix 4

Patient subpopulation-based results

Figure 1: Performances of different machine learning models on different patient subpopulations.

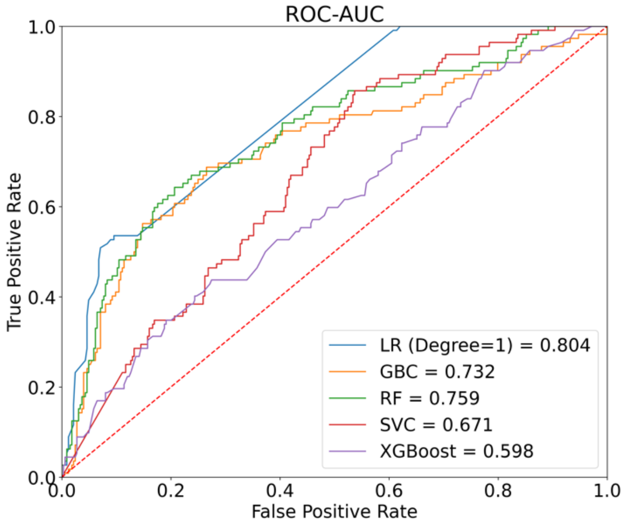

(a) Subpopulation of patients who were admitted to the hospital from an acute health care facility.

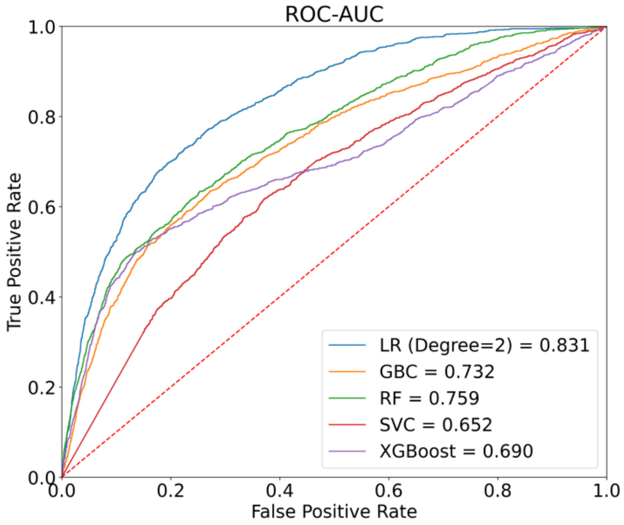

(b) Subpopulation of patients who were not admitted to the hospital from any acute health care facility.

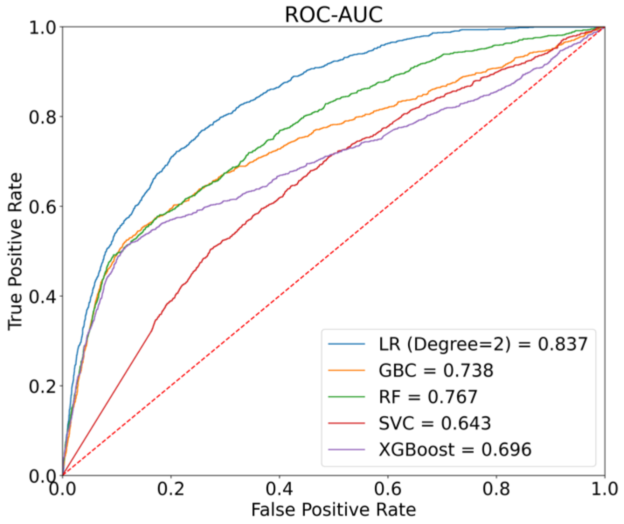

(c) Subpopulation of patients who were admitted to the hospital at most 15 days prior to test date.

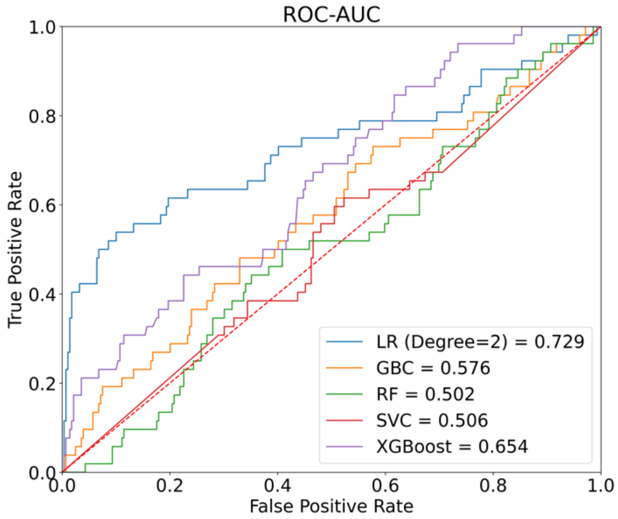

(d) Subpopulation of patients who were admitted to the hospital more than 15 days prior to test date.

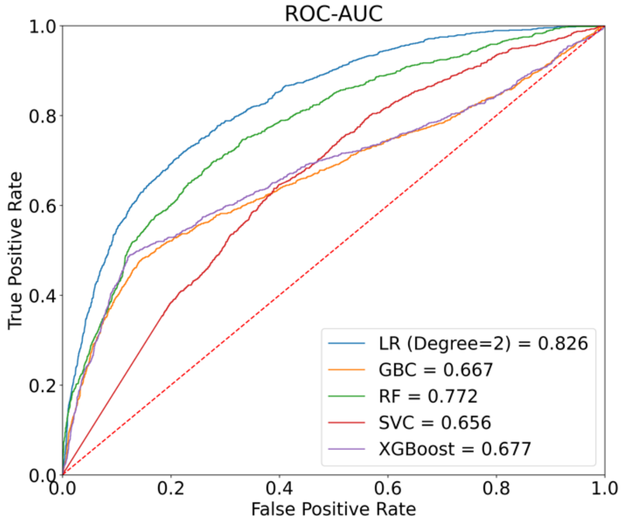

(e) Subpopulation of patients who were on antibiotic medications for at most 90 days.

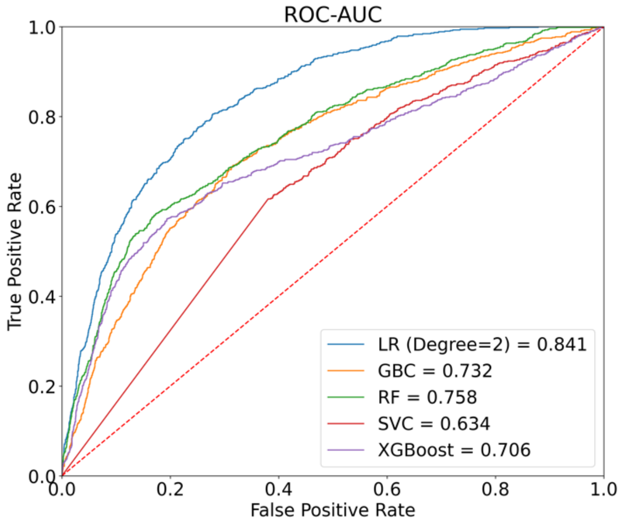

(f) Subpopulation of patients who were on antibiotic medications more than 90 days.

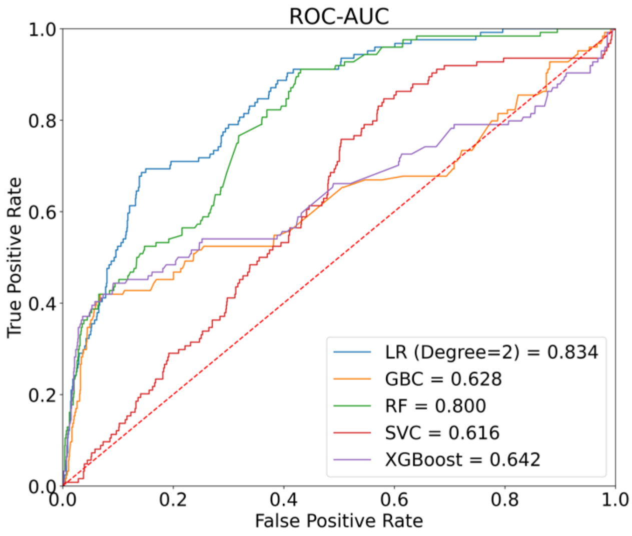

(g) Subpopulation of patients who did not consume any antibiotic medication.

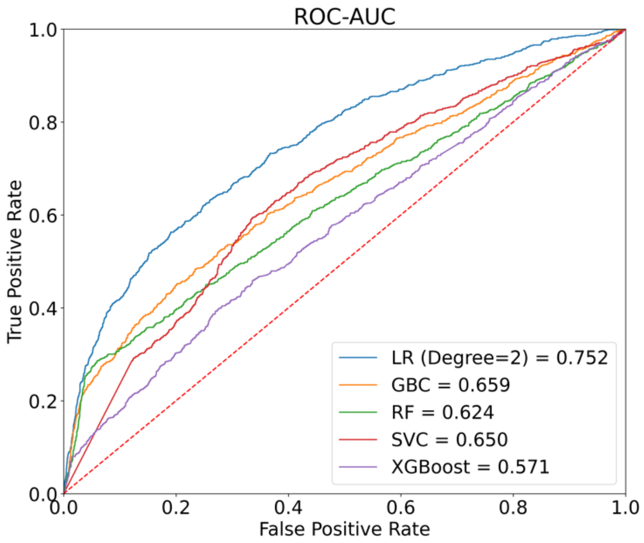

(h) Subpopulation of patients who did not say either in ICU or emergency department.

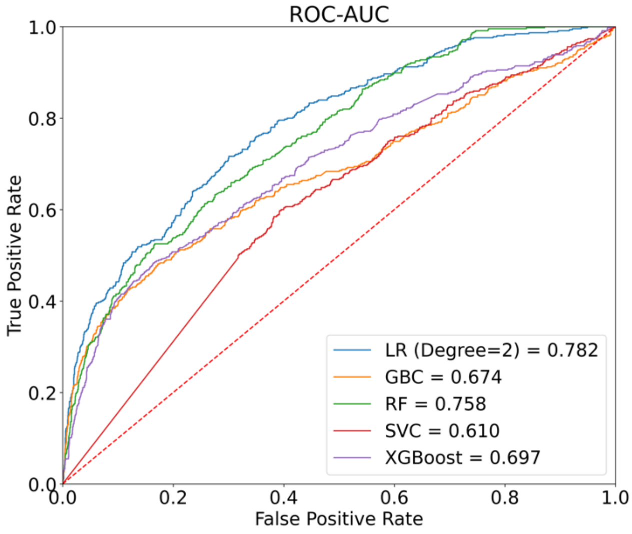

(i) Subpopulation of patients who are [0-50] years old.

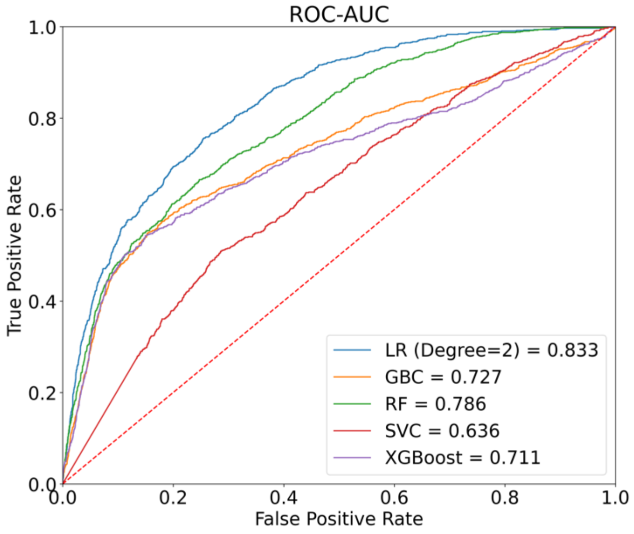

(j) Subpopulation of patients who are more than 50 years old.

Model hyperparameters

Table 1: The hyperparameters of the best-performing model for the patient subpopulations. The ‘Model’ column specifies the best-performing model. LR stands for the “Penalized logistic regression classifier”.

**Subpopulation**	**Model**	**Hyperparameter**
Overall	LR	C=0.01, penalty=l_1, degree=2
ICU	LR	C=0.01, penalty=l_1, degree=2
ED	LR	C=0.1, penalty=l_1, degree=2
Other rooms	LR	C=0.01, penalty=l_1, degree=2
From HCF	LR	C=0.01, penalty=l_1, degree=1
Not from HCF	LR	C=0.01, penalty=l_1, degree=2
Hosp. stay $\leq$15 days	LR	C=0.01, penalty=l_1, degree=2
Hosp. stay $>$ 15 days	LR	C=0.01, penalty=l_1, degree=2
Antibiotic $\leq$90 days	LR	C=0.01, penalty=l_1, degree=2
Antibiotic $>$ 90 days	LR	C=0.01, penalty=l_1, degree=2
No antibiotic	LR	C=0.01, penalty=l_1, degree=2
Age-group [0 −50]	LR	C=0.01, penalty=l_1, degree=2
Age-group 50^+	LR	C=0.01, penalty=l_1, degree=2
